# Nano-scale measurement of biomolecules by optical microscopy and semiconductor nanoparticles

**DOI:** 10.3389/fphys.2014.00273

**Published:** 2014-07-29

**Authors:** Taro Ichimura, Takashi Jin, Hideaki Fujita, Hideo Higuchi, Tomonobu M. Watanabe

**Affiliations:** ^1^Laboratory for Comprehensive Bioimaging, RIKEN Quantitative Biology CenterSuita, Osaka, Japan; ^2^Laboratory for Nano-Bio Probes, RIKEN Quantitative Biology CenterSuita, Osaka, Japan; ^3^Graduate School of Frontier Biosciences, Osaka UniversitySuita, Osaka, Japan; ^4^WPI, Immunology Frontier Research Center, Osaka UniversitySuita, Osaka, Japan; ^5^Department of Physics, School of Science, The University of TokyoBunkyo, Tokyo, Japan

**Keywords:** single particle tracking, super-resolution, fluorescent microscopy, quantum dot, nanoparticle

## Abstract

Over the past decade, great developments in optical microscopy have made this technology increasingly compatible with biological studies. Fluorescence microscopy has especially contributed to investigating the dynamic behaviors of live specimens and can now resolve objects with nanometer precision and resolution due to super-resolution imaging. Additionally, single particle tracking provides information on the dynamics of individual proteins at the nanometer scale both *in vitro* and in cells. Complementing advances in microscopy technologies has been the development of fluorescent probes. The quantum dot, a semi-conductor fluorescent nanoparticle, is particularly suitable for single particle tracking and super-resolution imaging. This article overviews the principles of single particle tracking and super resolution along with describing their application to the nanometer measurement/observation of biological systems when combined with quantum dot technologies.

## Introduction

Fluorescence microscopy has become standard for studying the dynamic behavior of biological phenomena such as the expression, movement, and localization of proteins and other molecules (Ellinger, 1940; Lichtman and Conchello, 2005; Drummen, 2012; Miyawaki, 2013; Peter et al., 2014). Optical diffraction, however, limits the spatial resolution to several 100 nanometers, denying information on many details about these phenomena (Abbe, 1873). Two technologies have since overcome this limitation and permit the observation of even smaller nano-scale dynamics: single particle tracking (Ritchie and Kusumi, 2003; Saxton, 2009; Chenouard et al., 2014) and super-resolution microscopy (Schermelleh et al., 2010; Galbraith and Galbraith, 2011; Leung and Chou, 2011). Single particle tracking pursues the position of single fluorescent probes conjugated to separate target proteins over a two-dimensional (2D) plane. Super-resolution microscopy, on the other hand, provides highly resolved optical images beyond the aforementioned spatial resolution.

To conduct the above imaging techniques, it is often required to label the target protein with a fluorescent probe. Fluorescent proteins are most popular for this purpose because of their simple and easy labeling procedure in live cells (Shimomura and Johnson, 1692; Tsien, 1998; Nifosí et al., 2007). Organic dyes are also common because of their wide application (Wombacher and Cornish, 2011; Wysocki and Lavis, 2011; Terai and Nagano, 2013). Another group of probes gaining attention is inorganic nanoparticles made of semiconductors, metals, silicon, etc. (Ruedas-Rama et al., 2012; Chinnathambi et al., 2014; Cupaioli et al., 2014). Although usually larger than fluorescent proteins and organic dyes, inorganic nanoparticles have generally stronger and more stable fluorescence profiles, which makes them applicable not only to basic research, but also to clinical studies (Byers and Hitchman, 2010; Choi and Frangioni, 2010; Saadeh et al., 2014; Wang and Wang, 2014). Furthermore, these same properties make them well suited for single particle tracking methods (Chang et al., 2008; Saxton, 2008; Barroso, 2011; Bruchez, 2011; Clausen and Lagerholm, 2011; Ruthardt et al., 2011; Pierobon and Cappello, 2012; Kairdolf et al., 2013; Petryayeva et al., 2013).

This review article focuses on advanced microscopy using quantum dots (Qdots), perhaps the most studied of inorganic nanoparticles for biological application (Pilla et al., 2012). Single particle tracking using Qdots has reached three dimensions (X, Y, Z) (Genovesio et al., 2006; Holtzer et al., 2007; Watanabe and Higuchi, 2007; Watanabe et al., 2007; Ram et al., 2008, 2012; Wells et al., 2008, 2010; Yajima et al., 2008), and more recently has even reached four dimensions (X, Y, Z, θ or X, Y, θ, φ) (Ohmachi et al., 2012; Watanabe et al., 2013). For all their benefits, Qdots do have drawbacks, however, including high blinking (Nirmal et al., 1996; van Sark et al., 2001; Schlegel et al., 2002; Hohng and Ha, 2004; Ko et al., 2011) and a spectral blue-shift during observation (Nirmal et al., 1996; van Sark et al., 2002; Hoyer et al., 2011), which complicate the continuous tracking of the single particle and emerge due to photo-oxidation while under high-power illumination. These limitations have stimulated research into new super-resolution microscopy methods (Lidke et al., 2005; Dertinger et al., 2009; Watanabe et al., 2010; Chien et al., 2011; Hoyer et al., 2011; Deng et al., 2014).

## Qdots as fluorescent labeling probes

**Figure 1:**
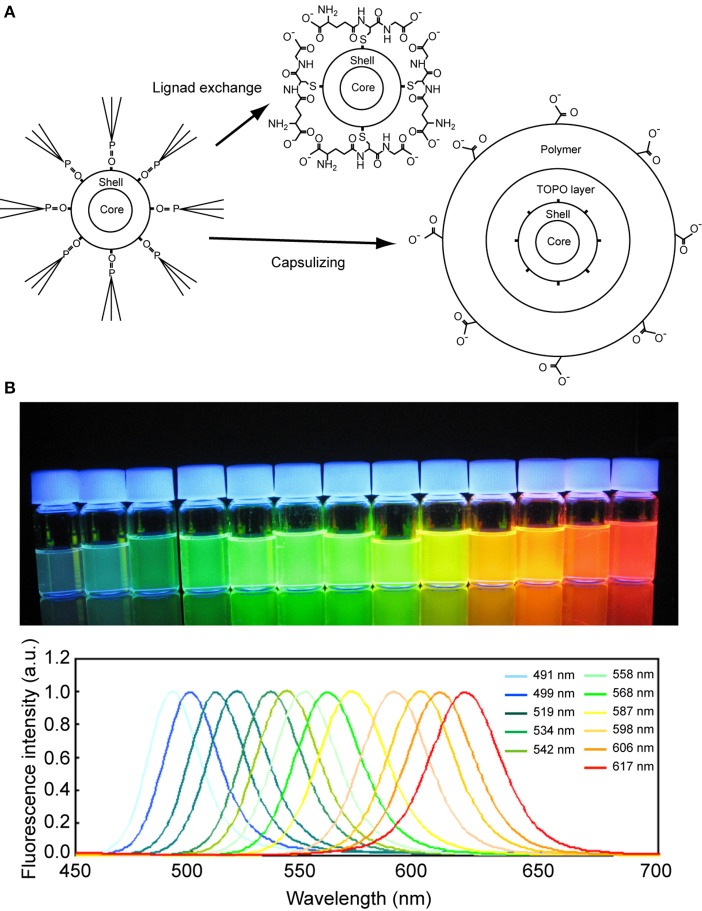
**Quantum dot. (A)** Schematic drawing of the surface modification of a Qdot. **(B)** Fluorescence photograph (upper) and spectra (lower) of Qdots of various diameters. The Qdots were excited by a UV light of 365 nm wavelength.

A Qdot is a semiconductor nanocrystal with electronic characteristics that depend on its size and shape (Rossetti et al., 1980; Ekimov and Onushchenko, 1981). Because of its unique characteristics and ease of synthesis, Qdots have been applied not only to biomedical research, but also to engineering- and industrial-related fields such as transistors, solar cells, LEDs, and diode lasers (Pilla et al., 2012). Qdots used in biological studies have a core-shell structure (Figure 1A); the most famous being the cadmium selenide (CdSe) core and zinc sulfide (ZnS) shell (Dabbousi et al., 1997; Bruchez et al., 1998; Chan and Nie, 1998; Pilla et al., 2012). This structure results in Qdots having narrow emission spectra but wide absorption spectra (Figure 1B). There are two important criteria when applying Qdots to biological studies: solubility and conjugating capability (Li et al., 2010). Highly fluorescent Qdots are usually synthesized in organic solvents in coordination with compounds such as tri-n-octylphosphine oxide (TOPO) or alkylamine. These compounds coat the Qdot, making it too hydrophobic to be dissolved in water. Therefore, further surface coating or exchange with hydrophilic compounds is needed for use in biological assay. Furthermore, upon becoming water soluble, the surface of the Qdot must have reactive groups such as amino and carboxyl chains in order for the Qdot to conjugate with the target biological sample. The surface coating contributes not only to the water-solubilization but also to the stabilization of the fluorescence of Qdot in water because the photophysical properties are well affected by the surface coating (Kuno et al., 1997; Kloepfer et al., 2005). Some surface coating methods suppress the blinking that is a drawback of Qdot (Hohng and Ha, 2004; Fomenko and Nesbitt, 2008; Mandal and Tamai, 2011; Zhang et al., 2013).

There are mainly two ways to prepare water-soluble Qdots (Figure 1A) (Erathodiyil and Ying, 2011; Zhang and Clapp, 2011). The first is to encapsulate a hydrophobic Qdot with an amphiphilic polymer or phospholipid (Dubertret et al., 2002; Gao et al., 2005; Li et al., 2010; Tomczak et al., 2013). The second is a ligand-exchange method in which the capping hydrophobic ligands are exchanged with hydrophilic ones (Gerion et al., 2001; Guo et al., 2003; Pinaud et al., 2004; Kim et al., 2005; Nann, 2005; Jiang et al., 2006; Dubois et al., 2007). While the water-solubilized Qdot obtained by the first method is more stable and suitable for commercialization, its size increases to about 20~40 nm, which risks steric hindrance against the function of the target protein (Li et al., 2010). The ligand-exchange method is inferior in stability, but is a simpler synthesis process and produces a smaller Qdot. The thin coating layer is another advantage of this method, as it reduces the risk of steric effects that could compromise the function of the protein upon conjugation with the Qdot.

Many coating agents exist for the ligand-exchange method. These include mercaptocarboxylic acid (Jiang et al., 2006), carbon disulfide (Dubois et al., 2007), thiosilanol (Gerion et al., 2001), dendrimer (Guo et al., 2003), peptide (Pinaud et al., 2004), phosphine oxide (Kim et al., 2005), and polyethylenimine (Nann, 2005). Coating agents can also sometimes functionalize Qdots for specific purposes. Examples include β-cyclodextrin for ion-sensing (Palaniappan et al., 2004), cyclodextrin for redox-active substrates (Palaniappan et al., 2006), and cyclodextrin thiol for pH-sensing (Cao et al., 2006). We usually use glutathione as the coating compound because of its easier preparation, which requires only the mixing of hydrophobic Qdots with an aqueous glutathione solution (Jin et al., 2008; Tiwari et al., 2009). Glutathione-coated Qdots have two reactive groups (amino and carboxyl) that enable easy conjugation with the target protein and show no cytotoxity (Tiwari et al., 2009). They can also be kept mono-dispersed in solution for 3 months after solubilization.

## Fluorescence microscopy for nano-scale measurements/observations

**Figure 2:**
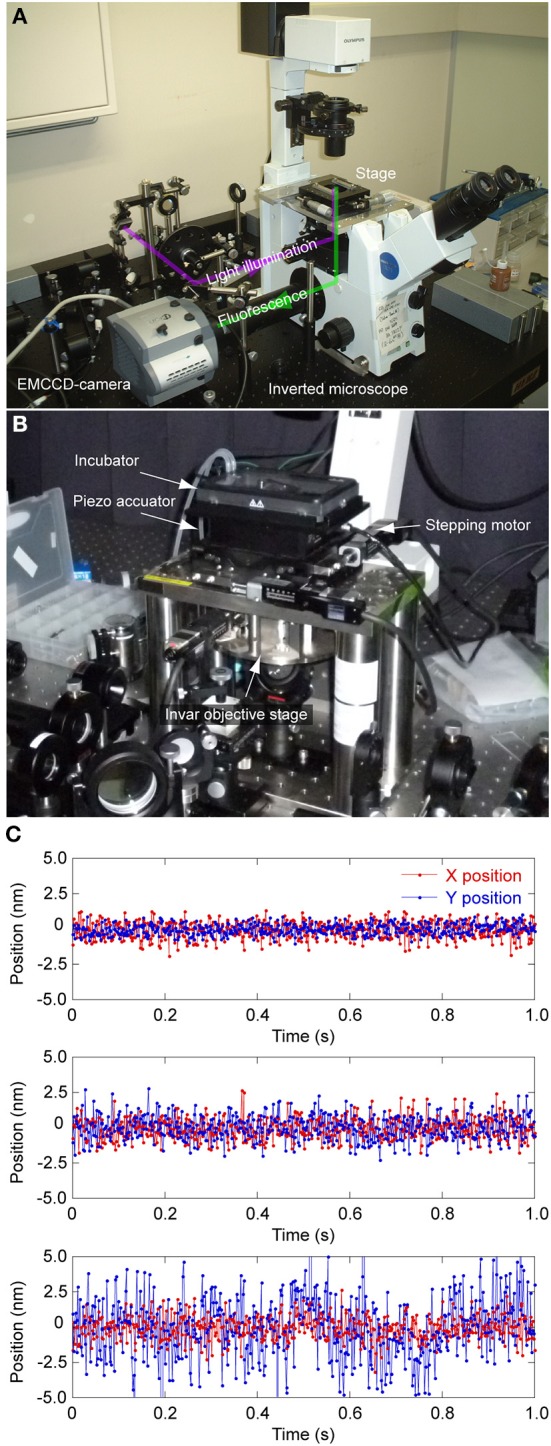
**Microscopic system for nanometery. (A)** Photograph of a typical microscopy setup. The system is mainly composed of an inverted fluorescent microscope (Olympus IX71), an objective lens (Olympus 60× PlanApo, 1.45 NA, oil immersion) and EMCCD camera (Andor iXon887 or 867). **(B)** The stage and objective revolver are made of duralumin and custom built. **(C)** Vibrations caused by different components of the microscope: all components rigidly fixed (upper), all components except the camera rigidly fixed (middle), and substitution of the mono-objective revolver with a 6-position revolver (lower). Red, X-position. Blue, Y-position. The stage position was determined by measuring the position of a glass bead absorbed on the sample surface.

The microscopy introduced in this review requires a regular wide-field fluorescence microscope and no complicated optical principles nor devices (Figure 2A). However, because nano-scale measurements require a high signal-to-noise ratio, a highly photon-sensitive camera, such as an electron multiplying charge coupled device (EMCCD) camera, is recommended. More recently, complementary metal-oxide-semiconductor (sCMOS) cameras have become available as alternatives (Huang et al., 2011; Long et al., 2012; Ma et al., 2013). The vibration and/or stage drift of the microscope should also be considered, as these can cause artifacts in the measurement by obscuring the behavior and structure of the target. Consequently, the microscope should be set on a vibration-isolation table and built with as minimal height and maximal rigidity as possible to decrease any vibration. Because thermal expansion of the metals composing the microscope causes drifts in the stage and focus position, microscopes made of metals with lower thermal expansion such as invar are generally preferred (Figure 2B). The drifts can be further suppressed by setting the microscopic system in a room with constant temperature and humidity.

Here we show one strategy for reducing vibrations. The transition images of a silica bead with 1 μm diameter absorbed on a coverslip surface were acquired with excess illumination so that the camera gain could be set to zero. The frame rate was 2.0 ms, the images were acquired for 1.0 s, and the precise position (X, Y) of the bead was calculated by image analysis. In our usual setup, the position of the bead was kept stable within 0.7 nm in the X-axis and 0.4 nm in the Y-axis (Figure 2C, upper). When a screw to fix the CCD camera was loosened, the vibration increased to 0.8 nm in both axes (Figure 2C, middle). Normally, we use a mono-objective revolver, but when instead a commercially-available 6-position revolver was used, we found the vibration enhanced in the Y-axis to 2 nm (Figure 2C, bottom). Thus, rigid construction of the microscope is paramount for nano-scale measurements and observations.

## Single particle tracking with nanometer precision using Qdots

**Figure 3:**
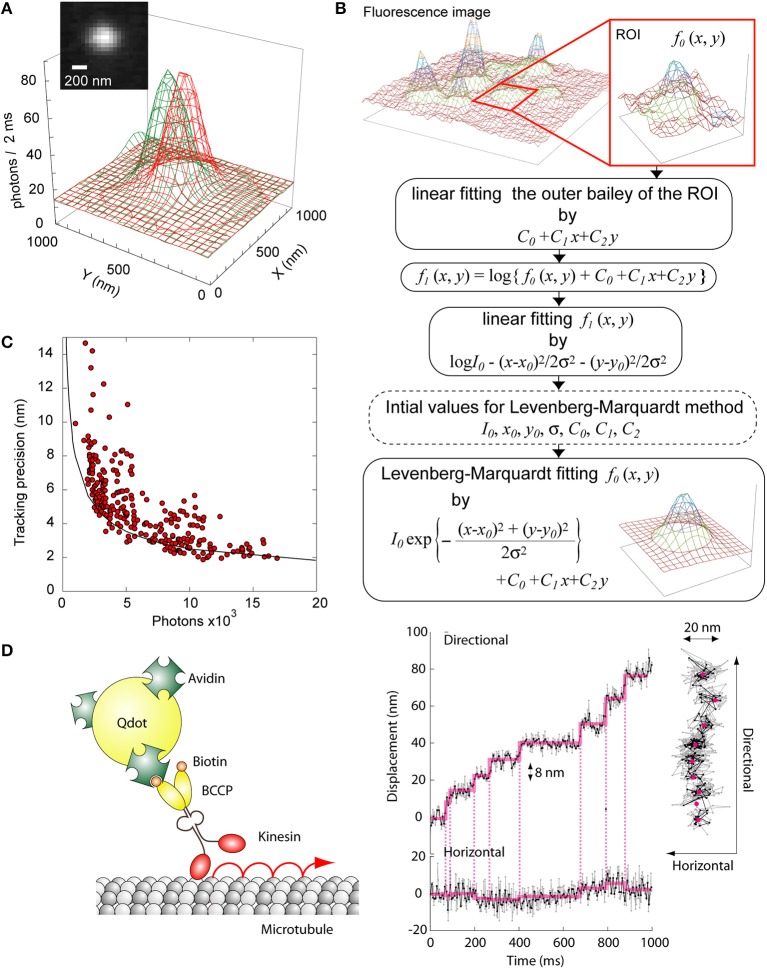
**Single particle tracking. (A)** Fluorescence profiles of a Qdot bound to a moving motor protein. The green-colored profile was taken at time 0 and the red profile at *t* = 2 s. The difference of the peaks of the two profiles was about 200 nm. (Inset) Fluorescent image of the Qdot. **(B)** Calculation scheme to determine the center position of a Qdot. **(C)** Relationship between the tracking precision by Gaussian fitting with the number of photons emitted from a single fluorophore. Circles, experimental data. Line, theoretical value (88). **(D)** Single particle tracking of kinesin. Left, schematic of the assay. Right, typical trace of a single kinesin. The frame rate of the image acquisition was 2 ms. The photon number emitted from the Qdot was about 10,000.

Single particle tracking is well applied for studies of motor proteins and membrane proteins, because resolving nano-scale movements is necessary for understanding the protein function (Ritchie and Kusumi, 2003; Park et al., 2007; Toprak and Selvin, 2007; Saxton, 2009). Although the resolution of conventional fluorescence microscopes is constrained by the diffraction limit, the 2D position of a single particle can be determined by calculating the weight center of the image of the fluorescent spot (Figure 3A). The fluorescence emitted from a fluorescent probe forms a point spread function (PSF) that can be fitted with a Gaussian distribution as

$$f\left(x,y\right)=I_{0}\cdot exp\left\{-\frac{{\left(x-x_{0}\right)}^{2}+{\left(y-y_{0}\right)}^{2}}{2\cdot \sigma^{2}}\right\}+C,$$

where *I*_0_ and (*x*_0_, *y*_0_) are the fluorescence intensity and the position of the fluorescing center, respectively, σ is the radial standard deviation of the Gaussian function, and *C* is the background fluorescence. This analysis can be used to measure the center position of the image (Kubitscheck et al., 2000; Cheezum et al., 2001; Thompson et al., 2002; Small and Stahlheber, 2014). Though there are other common methods for determining the center, including cross-correlation, sum-absolute difference, and simple centroid, Gaussian fitting has the highest robustness at low signal-to-noise ratios, which is common in biological studies (Thompson et al., 2002). In our case, the actual fitting computation is done by the Levenberg-Marquardt method (Levenberg, 1944). A practical example of our fitting is described below (Figure 3B). Because the background baseline is not always uniform in live-cell observations, we added additional parameters into the *C* term to fit the local background fluorescence with a tilted plane,

$$\begin{array}{l} f\left(x,y\right)=I_{0}\cdot exp\left\{-{\frac{{\left(x-x_{0}\right)}^{2}+\left(y-y_{0}\right)}{2\cdot \sigma^{2}}}^{2}\right\}\\ \;+C_{0}+C_{1}x+C_{2}y. \end{array}$$

This equation assumes the small area inside the region of interest (ROI) can be approximated by the plane. The initial parameters of the fitting are calculated by the linear least-square method for *C*_0_, *C*_1_, and *C*_2_ using only the outer boundary of the ROI. Because the logarithm of the subtraction between *f*(*x*_0_, *y*_0_) and *C*_0_ + *C*_1_*x* + *C*_2_*y* is a simple quadratic function, the other initial parameters are obtained by the linear least-square method, too. Setting the initial values close to the true values by these simple pre-calculations allows us to effectively reduce the number of the loop iterations in the Levenberg-Marquardt method.

The calculation precision by Gaussian fitting strongly depends on the photon number that the detection device receives from the emission of the fluorescent probe and can be as small as a few nanometers (Figure 3C) (Deschout et al., 2014; Small and Stahlheber, 2014). The method described above is called fluorescence imaging with one-nanometer accuracy (FIONA) and has quickly become a standard in the field (Yildiz et al., 2003; Yildiz and Selvin, 2005; Park et al., 2007; Hoffman et al., 2011). However, the number of photons emitted by single organic dyes and fluorescent protein molecules before photobleaching, about 110,000 (Kubitscheck et al., 2000), is too low for the observation of protein movement over a long time. Since the cause of photobleaching is thought to be oxygen collisions with the dye molecule in its excited state, it can be mitigated by the addition of oxygen scavengers (Sambongi et al., 1999; Adachi et al., 2000). Thus, the photon number from a single dye molecule can be increased to 1.4 million photons before photobleaching (Yildiz and Selvin, 2005). Meanwhile, Qdots show slight photobleaching and strong fluorescence even in the absence of scavengers (Bruchez et al., 1998). Though non-fluorescent nano-particles such as gold nano-particles are becoming increasingly popular for precise and long-term tracking using absorption (Kusumi et al., 2005; Lasne et al., 2006) or scattering (Nishikawa et al., 2010), the Qdot is still preferred in biological studies because of its wider color spectrum.

We investigated the relationship between the tracking precision and the average number of photons emitted from a Qdot (Figure 3C). The tracking precision was defined as the standard deviation of 100 data obtained with a Qdot immobilized on a glass surface in our case. While the experimental accuracy was a little lower than the theoretical expectation because of high blinking, it was still 2 nm when the photon number from a Qdot was 15,000 per exposure. To demonstrate the potential of single particle tracking as a biological tool, we measured the movement of kinesin, a microtubule-mediated motor protein (Figure 3D). The motor domain of the kinesin was fused with biotin career protein (BCCP) and conjugated with a Qdot via biotin-avidin affinity. The Qdot-labeled kinesin were then bound to microtubules adsorbed onto a cover slip. Upon adding 1 mM ATP, the Qdot was seen to move unidirectionally along the microtubule without detaching, which is consistent with kinesin using ATP to move (Figure 3D, left). The unidirectional movement of kinesin was composed of successive 8 nm steps (Figure 3D, right). Thus, FIONA using Qdots provides a simple quantitative measurement for nano-scale tracking of proteins at the single molecular level.

## Three-dimensional single particle tracking with nanometer precision using Qdots

The original FIONA only measured movement on a spatial plane, but has since been expanded to three spatial dimensions. For this purpose, a three-dimensional (3D) image under a microscope is obtained by scanning the objective lens along the focal axis with an actuator (Watanabe and Higuchi, 2007; Wells et al., 2008). This scanning, however, decreases the temporal resolution of the tracking. To solve this problem, 3D tracking methods without the objective scanning have been developed (Genovesio et al., 2006; Holtzer et al., 2007; Watanabe et al., 2007; Ram et al., 2008, 2012; Wells et al., 2010; Jia et al., 2014). Multifocal planes microscopy uses the difference of distinct optical pathways to estimate the Z-position by obtaining simultaneously the fluorescence intensities of several focal images (Toprak et al., 2007; Watanabe et al., 2007; Dalgarno et al., 2010; Juette and Bewersdorf, 2010; Ram et al., 2012). Similarly, 3D tracking using a photon-limited double-helix response system with a spatial light modulator, which has two twisting lobes along the optical axis of the image, results in a single fluorescent probe appearing as two fluorescent spots from which the Z-position can be determined (Pavani et al., 2009; Lew et al., 2010).

**Figure 4:**
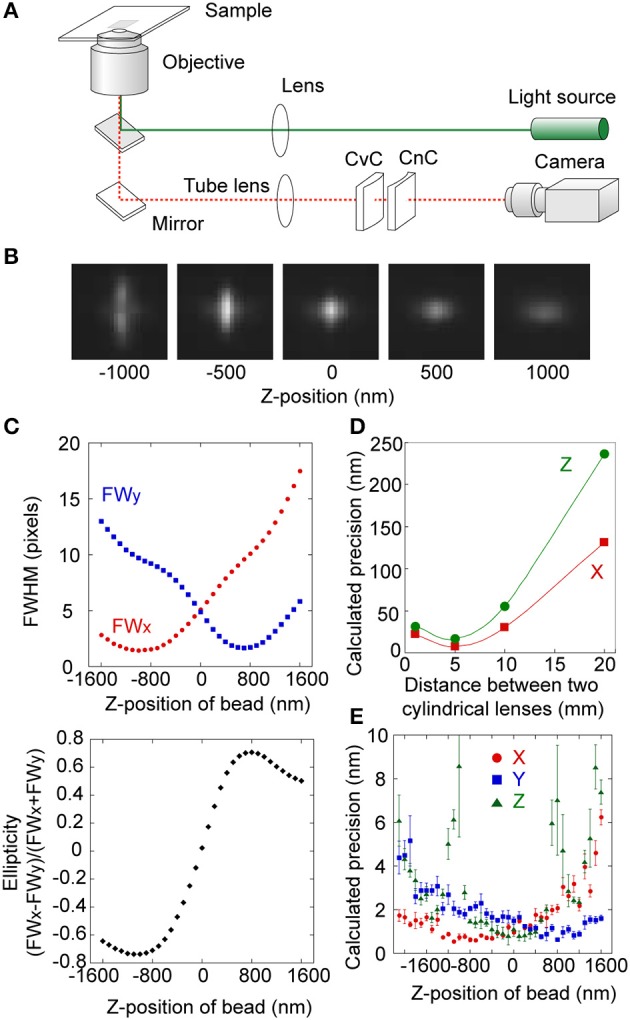
**3D single particle tracking using a pair of convex and concave cylindrical lens. (A)** Schematic drawing of the optical setup for 3D single particle tracking. CvC, convex cylindrical lens; CnC, concave cylindrical lens. **(B)** Fluorescent images of a single fluorescent bead with a diameter of 100 nm at various Z-positions (−1000 to 1000 nm). **(C)** Radial variances in the X-axis (FWx, red in upper panel) and Y-axis (FWy, blue in upper panel) of the Gaussian function, and ellipticity (lower panel) of a single fluorescent bead as a function of the Z-position (−1600 to 1600 nm). **(D)** Calculated precision in the X- (red) and Z-directions (green) as a function of distance between CvC and CnC. **(E)** Calculated precision in the X-, Y-, and Z-directions (red, blue, and green, respectively) as a function of Z-position when the camera received 15,000 photons from a fluorophore. The 3D tracking precisions was 2 nm in the X- and Y-axes and 5 nm along the Z-axis. Error bars represent standard deviations of 20 data.

One of the simplest 3D tracking methods intentionally generates astigmatism (Kao and Verkman, 1994; Holtzer et al., 2007; Izeddin et al., 2012). Here, a pair of convex and concave cylindrical lenses is inserted into the optical pathway before the detection device (Figure 4A) (Watanabe et al., 2013). These lenses generate different optical path lengths along the X- and Y-axes, resulting in a measurable relationship between the Z-position of the particle and the ellipticity of the PSF (Figure 4B). To calculate the ellipticity in addition to the 2D position, the below approximation formula is used

$$\begin{array}{l} f\left(x,y\right)=I_{0}\cdot exp\left\{-{\frac{\left(x-x_{0}\right)}{2\cdot \sigma_{x}^{2}}}^{2}\right\}\cdot exp\left\{-{\frac{\left(y-y_{0}\right)}{2\cdot \sigma_{y}^{2}}}^{2}\right\}\\ \;+C_{0}+C_{1}x+C_{2}y, \end{array}$$

where σ_x_ and σ_y_ are the radial standard deviations of the Gaussian function along the X- and Y-axes respectively. The ellipticity is defined as the ratio of the full width at half maximum (FWHM) of the 2D Gaussian in the X- and Y- axes due to the different focal lengths (Figure 4C). Changing the distance between the convex and concave cylindrical lenses permits astigmatism for optimal tracking resolution (Figure 4D). When the detection device received 15,000 photons from a fluorescent probe, we achieved 3D tracking with precisions of 2 nm in the X and Y-axes and 5 nm along the Z-axis (Figure 4E). However, a reliable range was limited between a field view of −800 and 800 nm (Figure 4D, lower and Figure 4E). This drawback is common in many 3D tracking methods. A new 3D tracking method based on Airy beams, however, overcomes this problem. Here, a diffraction free self-bending PSF is applied to a two-channeled detection system (Jia et al., 2014), and the Z-position is translated to the distance difference of the two X-positions of the two channels. This method elongates the dynamic range of 3D tracking to 3 μm. Regardless of the 3D tracking method, the key is to extract Z information from the XY projection.

## Four-dimensional single particle using polarized Qdots

**Figure 5:**
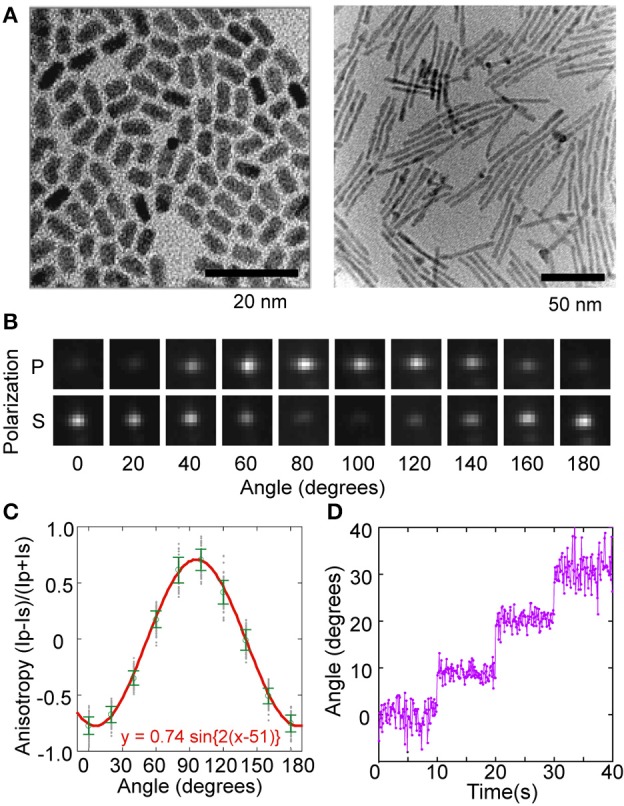
**Rod shaped quantum rods for single particle angular tracking. (A)** Transmission electron microscopy images of Odots (right) and Qrods (left). **(B)** Fluorescent images of a single Qrod on a coverslip acquired by simultaneously recording the P- (upper images) and S-polarization (lower images). **(C)** The anisotropy of a single Qrod as a function of the rotation angle. Gray, raw data; green circles, mean values; error bars, standard deviation. The mean values and standard deviations were calculated with 100 data points. The red line shows fitting with a sine function [y = a_1_· sin{2·(x- a_2_)}]. **(D)** Tracking of the artificial rotation steps. The Qrod was rotated stepwise 10 degrees every 10 s. The frame rate of the image acquisition was 100 ms. The photon number emitted from the Qrod was about 15,000.

As significant as acquiring the third spatial dimension is, 3D single particle tracking ignores any rotational movement made by the protein. To acquire the orientation, fluorescence anisotropy can be used, because the fluorescence emissions are of unequal intensities along the P and S polar axes (P- and S-polarization), which are defined by the polarizing beam-splitter, as described below (Werver, 1953; Albrecht, 1961; Harms et al., 1999). Anisotropy is defined as (*I*p−*I*s)/(*I*p+*I*s), where *I*p and *I*s are the intensities in P- and S-polarization, respectively (Harms et al., 1999). Anisotropy measurements have successfully tracked the rotatory dynamics of single protein molecules *in vitro* (Sase et al., 1997; Forkey et al., 2003) and in cells (Mizuno et al., 2011). The fluorescence anisotropy of a Qdot depends on the aspect ratio of its shape (Peng et al., 2000; Hu et al., 2001; Deka et al., 2009). Taking advantage of this property, a highly polarized rod-shaped Qdot (Qrod) can be synthesized by elongating the CdS shell along one-axis of the CdSe core (Figure 5A) (Peng et al., 2000; Hu et al., 2001). The anisotropy changes in Qrod fluorescence can be described as a sine function (Figures 5B,C) and the angular position by the arcsine function. The tracking precision of the orientation was about 1~2° when the photon number from a Qrod was 15,000 (**Figure 8D**). By utilizing this anisotropy technique, a fourth dimension, the angular (θ) component, could be added to the orthogonal 3 coordinate axes described by single particle tracking.

**Figure 6:**
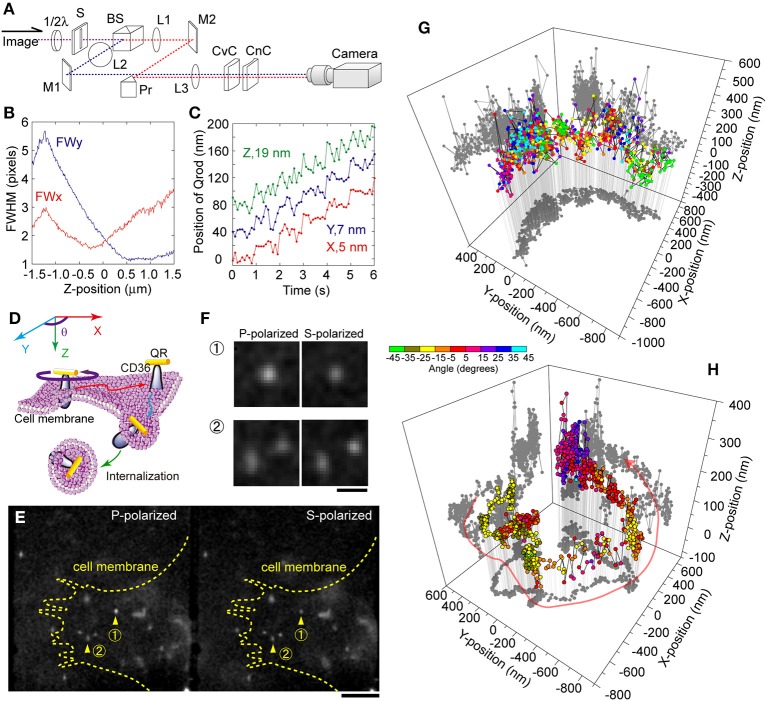
**4D single particle tracking using Qrod anisotropy. (A)** Schematic drawing of the optical setup for simultaneous 3D and angular single particle tracking. 1/2λ, 1/2-wave plate; S, slit; BS, beam splitter; L, lens; M, mirror; Pr, prism; CvC, convex cylindrical lens; and CnC, concave cylindrical lens. **(B)** FWHM values of the merged images of S- and P- polarized images as a function of the Z-position along the X- (red line) and Y-axes (blue line). **(C)** Tracking 20 nm steps when the Z-position of the Qrod was near zero. A Qrod fixed on a coverslip was moved at discrete 20 nm steps once every 1 s simultaneously along the X- (red), Y- (blue), and Z- axes (green). The frame rate of the image acquisition was 100 ms. Standard deviations of the tracking for 10 s (excluding the stepping moments) were 5, 7, and 19 nm along the three respective axes. The photon number emitted from the Qrot was about 1500. **(D)** Schematic depiction of the internalization of Qrod-labeled CD36, a membrane protein, from the cell membrane to the cytoplasm. **(E)** Fluorescent images of Qrods bound to membrane proteins in a living cell and simultaneously recorded in P- (left panel) and S-polarization (right panel). Arrowheads indicate two typical views that are enlarged in **(F)**. Scale bar is 5 μm. **(F)** Enlarged images of the spots marked by arrowheads in **(E)**. Scale bar, 1 μm. **(G,H)** Typical 4D traces of a single Qrod on the membrane **(G)** and near the nucleus **(H)**. The angle of the Qrod is indicated by the color bar.

In our 4D tracking system, a polarizing beam splitter is set before the cylindrical lens pair in the 3D tracking optics to divide the fluorescent image into S- and P- polar channels (Figure 6A) (Watanabe et al., 2013). For 3D tracking, the P- and S-polarized images are summed before calculating the 3D position. A small gap is generated if the two channels are not completely overlapped, leading to an asymmetrical relationship between the respective FWHM values of the X- and Y-axes (Figure 6B). The 3D position can be determined by fitting the merged PSF with a 2D Gaussian function, as mentioned above, and the orientation can be determined by the ratio of the intensities of the S- and P- polarized images. Thus, X, Y, Z, and θ are simultaneously obtained with an acquired image. In our case, when the number of photons from a single Qrod was about 10,000 and the Z-position was near zero, the calculated precisions for the X, Y, Z, and θ-positions were at maximum 5, 7, 9 nm and 1°, respectively (Figure 6C).

We used 4D tracking to observe the movement of a membrane protein conjugated with a Qrod via antibody affinity (Figures 6D–F) (Watanabe et al., 2013). Isolated Qrods moving on the membrane were identified under a fluorescence microscope (Figure 6E). The different intensities in the P- and S-polarized images indicated that the Qrod was inclined against the optical axis (Figure 6F, upper panels). One circular and one elliptical spot indicated that the two Qrods were at distinct Z-positions (Figure 6F, lower panels). One Qrod showed a half-moon like motion in the X- and Y-axes, which was accompanied by highly fluctuating movements along the Z-axis and fast rotational motion before endocytosis (Figure 6G). This observation suggests that this protein's lateral diffusion was constrained by the membrane undercoat, but that it could rotate freely along the plasma membrane. In the cytoplasm, a membrane protein seemed to be moving along tracks, most likely microtubules, in three-dimensions and slowly rotated helically (Figure 6H).

Another 4D tracking method was developed to obtain X, Y, θ, and φ coordinates, the last of which provides information on the out-of-plane tilt angle (Ohmachi et al., 2012). In this method, single Qrods are imaged as four crowded fluorescent spots by dividing the beam path using a beam splitter and two Wollaston prisms. Otherwise, the orientation of the individual fluorescent probe can be directly estimated using the dipole emission patterns of a defocused image (Bartko and Dickson, 1999a,b; Fourkas, 2001; Böhmer and Enderlein, 2003; Lieb et al., 2004), an approach that was successfully applied to the 4D tracking of a motor protein (Toprak et al., 2006). The combination of the Wollaston prism method with defocusing could achieve comprehensive tracking of all rotatory and translational movements of a biomolecule in a living cell.

## Super-resolution using blinking of Qdots

Super-resolution microscopy describes the resolution of two objects closer than the diffraction limit of light (Schermelleh et al., 2010; Galbraith and Galbraith, 2011; Leung and Chou, 2011). It can be classified into two main categories. The first is based on the photo-transition of a fluorescent probe between its radiative and non-radiative states in order to confine the fluorescence emission into a sub-diffraction-limit sized volume. This approach is known as RESOLFT (REversible Saturable OpticaL Fluorescence Transitions) and was first proposed and demonstrated by STED (STimulated Emission Depletion), which exploits the stimulated emission phenomenon of a fluorescent dye (Hell and Wichmann, 1994; Klar and Hell, 1999). RESOLFT can also be realized by other photoreactions, including those from a ground-state transition phenomenon (GSD: Ground State Depletion) (Hell and Kroug, 1995; Bretschneider et al., 2007), the saturation of fluorescence excitation (SAX: SAturated eXcitation) (Fujita et al., 2007), or from reversibly photoswitchable fluorescent proteins (Hofmann et al., 2005). RESOLFT can also be combined with structured illumination microscopy (SIM) (Heintzmann and Cremer, 1999; Gustafsson, 2000) to provide wide field imaging capability with superresolution (Heintzmann, 2003; Gustafsson, 2005).

The second category is based on the separate detection of individual single fluorescent probes in the time domain or spectra domain, and can be further decomposed into different concepts. One, known as SPDM (Spectral Precision Distance Microscopy), precisely localizes individual probes over the many frames of sequentially obtained images (Bornfleth et al., 1998; Lemmer et al., 2008). Stochastic optical reconstruction microscopy (STORM) (Rust et al., 2006) and fluorescence photoactivation localization microscopy (FPALM) (Betzig et al., 2006) are both SPDM-based techniques that utilize repeated activation-deactivation cycles of photoswitchable fluorophores such that the fluorescence spots on an obtained image are completely discrete.

Another method from the second category is blinking based superresolution (BBS). BBS relies on the randomness and non-Gaussian property of blinking, which means stochastic processing can be used to localize individual fluorescent probes. The first report of BBS used independent component analysis, which is a computational method that decomposes a multivariate signal into independent non-Gaussian signals (Lidke et al., 2005). Other BBS-based techniques use the temporal high-order cumulant (super-resolution optical fluctuation imaging: SOFI) (Dertinger et al., 2009), the temporal high-order variance (Variance Imaging for Superresolution: VISion) (Watanabe et al., 2010), spatial covariance (spatial covariance reconstructive: SCORE) (Deng et al., 2014), or Bayesian statistics (Cox et al., 2011). A great advantage of SPDM and BBS is that they need only a relatively simple fluorescent microscope and no complicated optics.

**Figure 7:**
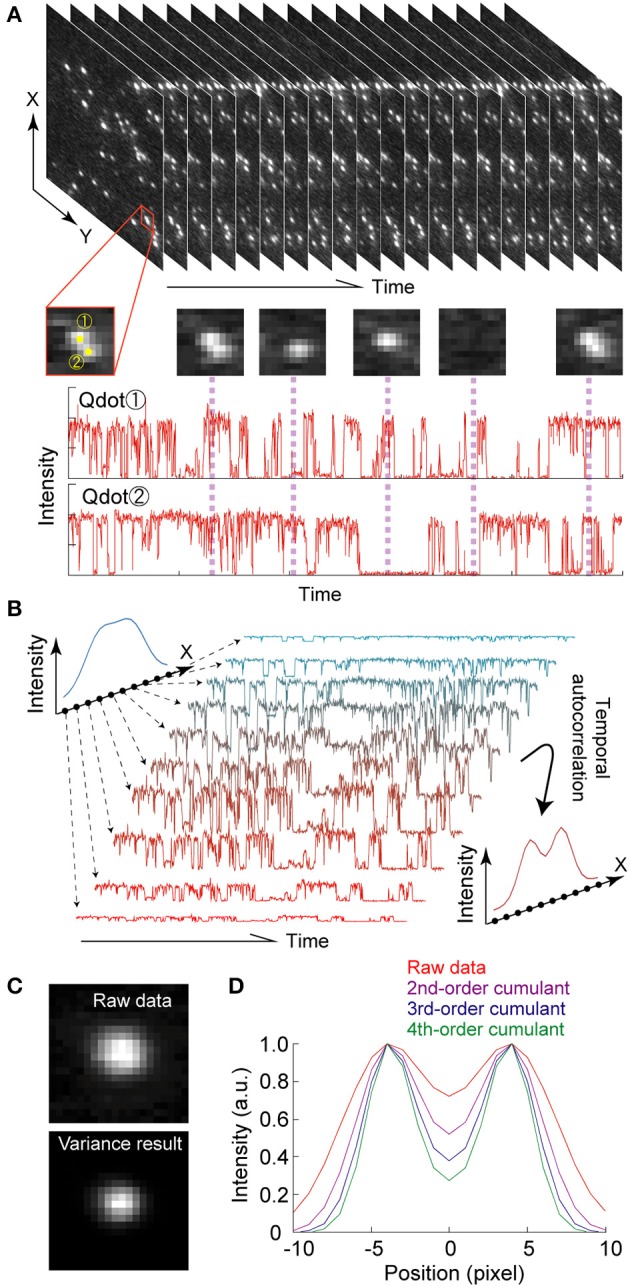
**Super-resolution using fluorescence fluctuations of a Qdot. (A)** Conceptual drawing super-resolution microscopy using fluorescence fluctuations of a Qdot. For details, see text. **(B)** Principle of SOFI. Each pixel contains a time trace, which is composed of the sum of the fluorescence from individual Qdots. Calculating the temporal autocorrelation of each pixel gives a new image whose spatial resolution is improved. **(C)** Fluorescent image (upper) and 2nd autocorrelation (equal to variance; lower) image of a single Qdot. Pixel size, 50.4 nm. **(D)** Point spread functions in one-dimension of the SOFI simulation results. In the simulation, the two Qdots were set at distance of 4 pixels apart. The FWHM of each Qdot was also 4 pixels. 2500 images were used.

Qdots are the most compatible with BBS owing to their strong blinking phenomenon. Supposing that there are two adjoining Qdots independently and randomly fluctuating, the moment that one Qdot emits and the other does not is a stochastic event (Figure 7A). As an example, a solution to identifying the Qdot for SOFI and VISion is shown below (Dertinger et al., 2009). A fluorescent image of Qdots, *F*(***r***,*t*), is expressed by the convolution of a PSF, *U*(***r***), of the optical system and the brightness, ε*_k_s_k_*(*t*), where ***r****_k_*, ε*_k_*, and *s_k_*(*t*) are the position and the time-invariant and -variant components of brightness of the *k*-th Qdot, respectively.

$$F\left(r,t\right)=\sum_{k}U(r-r_{k})\cdot \varepsilon_{k}\cdot s_{k}(t)$$

The second-order autocorrelation function, *G*_2_(***r***,τ), is then given by *F*(***r***,*t*) as follows,

$$\begin{array}{l} G_{2}\left(r,\tau \right)={\langle \delta F\left(r,t+\tau \right)\cdot tF\left(r,t\right)\rangle }_{t}\\ \;=\sum_{j,k}U\left(r-r_{j}\right)\cdot U\left(r-r_{k}\right)\cdot \varepsilon_{j}\cdot \varepsilon_{k}\cdot \\ \;{\langle \delta s_{j}\left(r,t+\tau \right)\cdot ts_{k}\left(r,t\right)\rangle }_{t}\\ \;=\sum_{k}U^{2}(r-r_{k})\cdot \varepsilon_{k}{}^{2}\cdot {\langle \delta s_{k}\left(r,t+\tau \right)\cdot ts_{k}\left(r,t\right)\rangle }_{t} \end{array}$$

where <···>_t_ and δ(·) denote a time-averaging operation and deviation from the time-average, respectively. Because of the independency of the fluorescence fluctuation of the two distinct Qdots (*k* ≠ *j*), the time average of their product is zero. For simple comparison of the raw image, *F*(***r***,*t*), and the auto-correlation image *G*_2_(***r***,*τ*), we here substitute 0 for the delay time, τ, to reduce *G*_2_(***r***,*τ*) to *G*_2_(***r***,0).

$$G_{2}\left(r,0\right)=\sum_{k}U^{2}(r-r_{k})\cdot \varepsilon_{k}{}^{2}\cdot {\langle \delta s_{k}^{2}\left(t\right)\rangle }_{t}$$

This equation indicates that G_2_(**r**, τ) is given by the convolution of *U*^2^(*r*) and the square of ε_k_s_*k*_(t). Assuming that *U*(***r***) is approximated by a Gaussian distribution, the spatial resolution of *G*_2_(***r***,*0*) is improved v2 times from *F*(***r***,*t*), but at the expense of temporal information, since the spatial resolution of the optical microscopic image is limited by the sharpness of the PSF (Figures 7B,C). The higher-order autocorrelation contains high-spatial frequency information. However, because this is a moment value that contains cross-terms from the lower-order correlation contributions, the accrual spatial resolution for distinguishing two Qdots cannot be improved more than √2 times. It is therefore necessary to transform the *n*th-order correlation into an *n*th-order cumulant that consists only of terms containing the *n*th power of the PSF. While the higher order cumulant gives higher spatial resolution (Figure 7D), a huge number of images are still needed.

**Figure 8:**
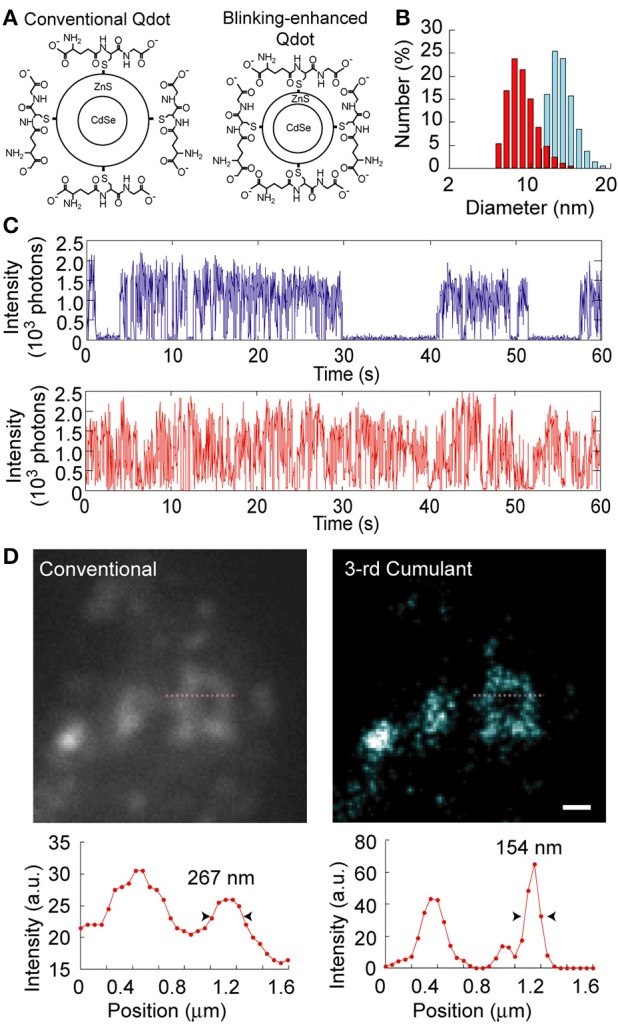
**Super-resolution with blinking-enhanced Qdots. (A)** Schematic drawing of blinking-enhanced Qdots, which were achieved by making the ZnS shell thinner. **(B)** Diameter of a commercial Qdot (blue) and the developed Qdot (red) coated with glutathione. **(C)** Time courses of the fluorescence intensities of the commercial (upper) and developed Qdots (lower). The illuminating laser power was regulated to keep the average fluorescent intensities of the two Qdots approximately the same. **(D)** Super-resolved image using the developed Qdot and SOFI. Upper panels show vesicles labeled with the developed Qdot under a conventional fluorescence microscope (left) and SOFI (3rd cumulant image using 100 images) (right) imaging. Pixel size, 50.4 nm. White scale bar, 500 nm. Lower panels show the intensity profiles of the one-dimension cross-sections (magenta lines) in the upper panels. The arrowheads indicate the spatial resolution.

To decrease the required number of images, we developed a highly fluctuating Qdot in which the switching frequency between the on- and off-state was greatly increased by optimizing the shell thickness to promote more interaction between the CdSe-core and oxygen atoms in water (Figures 8A,B). Though the quantum yield of this Qdot was less than that of standard Qdots, it still had sufficient intensity and stability when exposed to high power illumination, and no long off-state was observed (Figure 8C). Hence, we could easily obtain a super-resolved image by only labeling the target protein and calculating the fluctuation of the blinking-enhanced Qdots (Figure 8D). In our case, the spatial resolution was improved from 267 to 154 nm using SOFI and only 100 images (Watanabe et al., 2010).

## Conclusion

Conventional optical microscopy can quantitatively acquire 3D position and orientation information at the nano-scale from the shape of the PSF and the polarization characteristics of Qdots and Qrods. The amount of spatial information can be increased by analyzing the stochastic fluctuations of the fluorescence. Thus, the fluorescence of a probe attached to a molecule can reveal information about the molecular phenomena and/or state. Increasing the intensity, stability, and blinking of Qdots and its derivatives will make the acquisition of such information even more feasible.

Super-resolution microscopy and single particle tracking have made it possible to resolve and follow two objects closer than the diffraction limit of light. The result is quantitative information of the dynamics of biological phenomena at the nano-scale. Even more details of the dynamics can be acquired with the above technologies by using Qdots and their derivatives as probes for labeling the molecules of interest. The PSF and the polarization characteristics of the Qdots can be used to provide comprehensive information on both the position and orientation of the molecule of interest. Because this information can be extracted from the stochastic properties of the fluorescence, increasing the intensity, stability, and blinking of Qdots should provide even more quantitative details about the dynamics.

### Conflict of interest statement

The authors declare that the research was conducted in the absence of any commercial or financial relationships that could be construed as a potential conflict of interest.
